# Prevalence of colibacillosis in chickens in greater Mymensingh district of Bangladesh

**DOI:** 10.14202/vetworld.2017.29-33

**Published:** 2017-01-11

**Authors:** Md. Abdul Matin, Md. Ariful Islam, Mst. Minara Khatun

**Affiliations:** Department of Microbiology and Hygiene, Faculty of Veterinary Science, Bangladesh Agricultural University, Mymensingh 2202, Bangladesh

**Keywords:** chicken, colibacillosis, multidrug resistant *Escherichia coli*, Mymensingh, Tangail

## Abstract

**Aim:**

This study was conducted for determination of the prevalence of colibacillosis in chicken in poultry farms in Mymensingh and Tangail districts. Isolation, identification, and antibiogram profile of *Escherichia coli* were also performed.

**Materials and Methods:**

A total of 25 chickens manifested clinical signs of colibacillosis were collected from five different poultry farms during natural outbreaks.

**Results:**

In broiler, the prevalence of colibacillosis was 0.84%, and in layer, prevalence was 0.80%. The prevalence of colibacillosis was 1.0% and 0.5% in 25-30 days old and 31-35 days old broiler, respectively. In case of layer birds, the prevalence was 0.6% in 40-45 days old bird and 1% in 46-50 days old bird. Identity of the *E. coli* isolate of chicken was confirmed by sugar fermentation, biochemical tests, and polymerase chain reaction assay. Antibiogram profile of *E. coli* isolate of chicken revealed that it was multidrug resistant (resistant against two antibiotics, such as ampicillin and cefalexin).

**Conclusion:**

Data of this study suggest that colibacillosis is prevalent in the study areas which underscore the need of implementation of prevention and control measure against this disease.

## Introduction

Avian pathogenic *Escherichia coli* (APEC) is the major cause of colibacillosis in poultry production [1]. It is a common disease in poultry flocks worldwide especially in the intensive farming system [2]. It affects birds of all ages. This disease has an important economic impact on poultry production worldwide [3]. It is one of the most common causes of mortality in commercial layer and breeder chickens [4]. Signs in birds affected with colibacillosis vary from sudden death to birds being off-color with their necks pulled into their bodies [5]. APEC strains are the etiologic agents of colibacillosis in birds [6].

*E. coli* is considered a member of the normal microflora of the poultry intestine, but certain strains, such as those designated as APEC, spread into various internal organs and cause colibacillosis characterized by systemic fatal disease [7-9]. Avian colibacillosis was found widely prevalent in all age groups of chickens (9.52-36.73%) with especially high prevalence rate in adult layer birds (36.73%) [10].

Avian colibacillosis is communicable to humans [11]. Although most strains of *E. coli* are not regarded as pathogens, they can be opportunistic pathogens that cause infection in immunocompromised hosts. There are also pathogenic strains of *E. coli* that when ingested, causes gastrointestinal illness in healthy humans and animals.

Treatment of food-producing animals without conducting sensitivity test is a common practice by non-veterinarians. And because there are no strict regulations guiding the use of antibiotics in food-producing animals in Bangladesh, farmers often use different antibiotics as growth promoters/prophylaxis in brooding their birds [12]. This scenario has led to selection pressure and development of multidrug resistance among *E. coli* isolates. This, in turn, has resulted to the increased virulence of *E. coli* and consequently to high rate of treatment failures and heavy economic losses often experienced by farmers [12].

Colibacillosis in chicken is endemic in Bangladesh [13]. Intensification of poultry production and the quick expansion of free-range production systems will increase the incidence of colibacillosis through greater exposure of birds to pathogens and stress [14]. Several studies such as occurrence, pathological investigation, and antibiotic sensitivity assay of colibacillosis of chicken have been performed in Bangladesh [15-18]. However, there is no comprehensive study of colibacillosis in broiler and layer birds in greater Mymensingh district of Bangladesh. The objectives of this study were (i) to isolates and identify *E. coli* from dead and sick birds by conventional bacteriological method and polymerase chain reaction (PCR), (ii) to determine the prevalence of colibacillosis on the basis of age, type and location, and (iii) to determine antibiogram profile of *E. coli* isolated from clinical cases of colibacillosis against five commonly used antibiotics.

## Materials and Methods

### Ethical approval

The experiment was approved by Institutional Animal Ethics Committee, Faculty of Veterinary Science, Bangladesh Agricultural University, Mymensingh 2202, Bangladesh.

### Study period

This study was conducted during the period ranging from July to December 2013.

### Collection of samples

Sick (n=6) and dead (n=19) birds manifesting the characteristics clinical signs of colibacillosis (watery diarrhea, weakness, anorexia and weight loss, etc.) were collected from five poultry farms in Mymensingh and Tangail districts (Table-1). Feces samples of sick birds were collected aseptically in sterile test tubes. Sick birds were euthanized by intracardiac administration of saturated MgSO_4_ solution. A thorough post-mortem examination of all dead birds was carried out. Liver and spleen samples were collected separately in sterile test tubes. Feces, liver, and spleen samples were used for bacteriological study (Table-2).

**Table-1:** Summary of chicken samples and study areas.

Name of farm	Number of dead bird	Number of sick bird	Total	Grand total
BAU broiler farm	6	1	7	25
CP broiler farm	2	0	2	
Abu Tarek broiler farm	5	1	6	
Nahar broiler farm	4	2	6	
Sotota layer farm	2	2	4	

BAU=Bangladesh Agricultural University

**Table-2:** Summary of clinical specimens.

Name of specimen	Number of samples examinated
Liver	25
Spleen	25
Faeces	25

Age (broiler 25-30 days and 31-35 days old and layer 40-45 days and 46-50 days old), chicken line and location of the farms were recorded.

### Isolation of bacteria in pure culture

Samples were enriched in nutrient broth at 37°C for 24 h. The overnight bacterial broths were streaked onto eosin methylene blue (EMB) agar and incubated at 37°C for 24 h. Single colony was further sub-cultured until a pure culture was obtained.

### Identification of bacteria

Colony characteristics of bacteria such as shape, size, surface texture, edge, elevation and color observed in pure culture, Gram’s-staining and biochemical tests (sugar fermentation, methyl red, Voges-Proskauer and Indole production tests) were used for identification of bacteria [19].

### Molecular detection of *E. coli* by PCR

#### DNA extraction

A pure bacterial colony was mixed with 100 µl of distilled water which was boiled for 10 min then immediately kept on ice for cold shock. Finally, centrifugation was done at 10,000 rpm for 10 min. The supernatant was collected and used as DNA template for PCR.

#### Primers used for PCR

A genus-specific PCR was performed to amplify 16S rRNA of *E. coli* using previously published primers [20]. The list of primer is furnished in Table-3.

**Table-3:** PCR primers with sequence.

Primer	Sequence	Size - (bp)
*E. coli* 16S (F)	5’- AATTGAAGAGTTTGATCATG-3’	704
*E. coli* 16S (R)	5’- CTCTACGCATTTCACCGCTAC-3’	

F=Forward, R=Reverse, bp=Base pair, PCR=Polymerase chain reaction

#### Antibiotic sensitivity test

Three isolates randomly selected from the samples were tested for antimicrobial drug susceptibility against five commonly used antibiotics such as ampicillin (10 µg/disc), chloramphenicol (30 µg/disc), ciprofloxacin (5 µg/disc), gentamicin (10 µg/disc), and cefalexin (30 µg/disc) by disk diffusion or Kirby-Bauer method [20]. Results of antibiotic sensitivity tests were recorded as sensitive and resistant according to the guidelines of Clinical and Laboratory Standards Institute [21].

### Statistical analysis

The prevalence of colibacillosis in broiler and layer at different age groups of bird and poultry farm compared for statistical significance using Chi-square test (Duncan’s multiple range test, SPSS 11.5, UK). A p≤0.05 was considered to be statistically significant.

## Results

### Isolation of *E. coli* on EMB media

The growth of *E. coli* on EMB agar was indicated by smooth, circular, black color colonies with metallic sheen.

### Identification of *E. coli*

*E. coli* on EMB agar produced greenish black colony with metallic sheen. On MacConkey agar it produced bright, pink colored, transparent smooth and raised colonies. Pink colored, rod-shaped, short chain, single or paired Gram-negative bacilli were observed after Gram’s staining. It fermented five basic sugars such as dextrose, sucrose, lactose, maltose, and mannitol with the production of both acid and gas. Acid production was indicated by the color change from reddish to yellow, and gas production was noted by the presence of gas bubbles in the inverted Durham’s tubes. All *E. coli* isolates were catalase, indole, M-R positive, and V-P negative.

### Overall prevalence of colibacillosis in chicken

The overall prevalence of colibacillosis was 0.84% in broiler and 0.80% in layer (Table-4).

**Table-4:** Overall prevalence of colibacillosis in broiler and layer.

Chicken lines	Flock size	Number of sick and dead birds	Overall prevalence (%)
Broiler	2500	21	0.84
Layer	500	4	0.80

### Age wise prevalence of colibacillosis in chicken

The prevalence of colibacillosis in broiler and layer on the basis of age is shown in Table-5. The prevalence of colibacillosis was 1% in 25-30 days old broiler and 0.5% in 31-35 days old broiler. On the other hand, prevalence was 0.6% in 40-45 days old layer and 1% in 46-50 days old layers. Prevalence of colibacillosis in broiler and layer at different age groups was statistically significant (p=0.006).

**Table-5:** Prevalence of colibacillosis in 25-30 days and 31-35 days old broiler and 40-45 days and 46-50 days old layer.

Chicken line	Age group	Flock size	Number of sick and dead birds	Prevalence (%)
Broiler	25-30 days old	1500	16	1.0
	31-35 days old	1000	5	0.5
Layer	40-45 days old	300	2	0.6
	46-50 days old	200	2	1.0

### Farm wise prevalence of colibacillosis in chicken

The farm wise prevalence of colibacillosis was 0.7% in Bangladesh Agricultural University (BAU) broiler farm, 0.4% in CP broiler farm, 1.2% in Abu Tarek broiler farm, 1.2% in Nahar broiler farm, and 0.8% in Sotota layer farm (Table-6). The prevalence of colibacillosis in all farms was found to be statistically significant (p=0.0054).

**Table-6:** Farm wise prevalence of colibacillosis.

Name of poultry farm	Flock size	Number of sick and dead birds	Prevalence (%)
BAU broiler farm	1000	7	0.7
CP broiler farm	500	2	0.4
Abu Tarek broiler farm	500	6	1.2
Nahar broiler farm	500	6	1.2
Sotota layer farm	500	4	0.8

BAU=Bangladesh Agricultural University

### Molecular detection of *E. coli*

**Figure-1:**
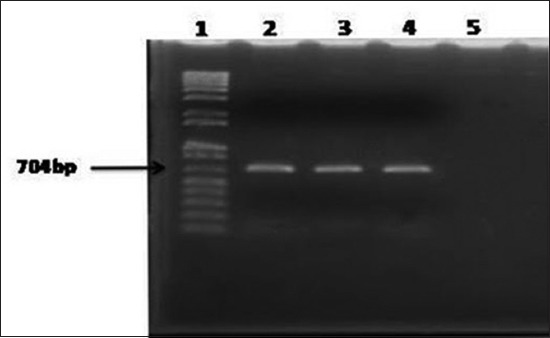
Results of polymerase chain reaction for amplification of 16S rRNA of *Escherichia coli* isolates of chicken. Lane 1: 100 bp-12 kb size DNA marker (Trackit, Invitrogen, USA); 2: DNA of *E. coli* isolate from liver; 3: DNA of *E. coli* isolates from spleen; 4: DNA of *E. coli* isolate from feces; 5: Negative control without DNA.

DNA extracted from three *E. coli* isolates were used in the PCR assay. PCR primers targeting 16S rRNA of *E. coli* amplified 704 bp fragments of DNA confirming the identity of *E. coli* (Figure-1).

### Results of antibiotics sensitivity tests

**Figure-2:**
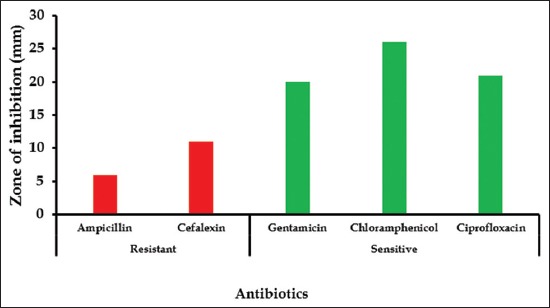
Zone of inhibition of ampicillin, cefalexin, gentamicin, chloramphenicol and ciprofloxacin against *Escherichia coli*.

On the basis of zone of inhibition *E. coli* isolates were found to be sensitive against ciprofloxacin, gentamicin and chloramphenicol and resistant against ampicillin and cefalexin (Figure-2).

## Discussion

Avian colibacillosis caused by *E. coli* is a major health problem in poultry industry [2]. In this study, an attempt was undertaken for determination of prevalence of colibacillosis in broiler and layer chicken from field outbreaks. In this study, chicken clinically infected with colibacillosis manifested characteristics clinical sign of colibacillosis such as watery diarrhea, anorexia, weakness, and loss of body weight. A similar type of clinical signs in colibacillosis was also recorded [22]. This study recorded 0.84% prevalence of colibacillosis in broiler and 0.80% in layer chicken. Higher prevalence of colibacillosis was found in broiler chicken (87.5%) than layer (76.04%) [23].

Prevalence of colibacillosis was recorded 28% in Sonali chicken on smallholder households in Bangladesh [13]. 5.51% prevalence of colibacillosis was reported in chicken in Mymensingh district of Bangladesh [24]. A study found 20.9% colibacillosis in chicken in small or medium scale commercial poultry farms in Mymensingh and its neighboring district [25]. 10.61% colibacillosis was recorded in chicken at the Central Disease Investigation Laboratory, Dhaka [26]. A study conducted in South Korea recorded (32.6%) prevalence of colibacillosis in broiler flock and 27.2% prevalence in layer flock [18]. In Pakistan, 8.9% colibacillosis was recorded in poultry farms around Faisalabad [27].

This study recorded 1% and 0.5% prevalence of colibacillosis in broiler belonged to 1 month and more than 1 month age groups chicken. In case of layer, this study recorded 0.6% prevalence up to 45 days old layer and 1% prevalence up to 50 days old layer. 9.52-36. 73% colibacillosis was found in all age group of bird [10]. The highest prevalence of colibacillosis was recorded in adult layer (36.73%) [10], but this study recorded lower prevalence in 40-45 days old layer (0.6%) as compared to 46-50 days old layer (1%).

In this study, EMB agar was used for isolation of *E. coli* from clinical samples. Colony characteristics of *E. coli* observed on EMB agar were similar to the findings [28]. Morphologically, *E. coli* were Gram-negative short rod arranged in single or paired and motile. Another researcher also described similar cultural and staining characteristics of *E. coli* [29]. The fact that pure cultures of *E. coli* were isolated from all the cultured samples established the incidence of colibacillosis in the farm [4]. The identity of *E. coli* spp. was confirmed by sugar fermentation and biochemical tests. In this study, PCR assay using specific primers amplified 704 bp fragment of 16S rRNA gene of *E. coli* isolates of chicken. Similar results were also observed by another researcher [30].

In this study, *E. coli* isolates of chicken were found to be resistant against two antibiotics such as ampicillin and cefalexin. Resistant profile of *E. coli* isolate suggested that they are multi-drug resistant. In another study, *E. coli* isolate of chicken was resistant to ampicillin [31]. *E. coli* isolate of chicken were also resistant to ampicillin [32,33]. Multi-drug resistant *E. coli* was reported in the healthy broiler chicken [34].

Antimicrobial therapy is an important tool in reducing huge losses in poultry industry caused by *E. coli* infection [35]. In this study, *E. coli* isolate of poultry was found sensitive to gentamicin, chloramphenicol, and ciprofloxacin. These antibiotics may be used for the treatment of clinical cases of colibacillosis of chicken. Antibiotic sensitivity profile of *E. coli* isolate of chicken recorded in this study is in agreement with the findings of others [31,32]. The current resistance phenotypes with MDR are similar to data reported from other researchers [36-38].

## Conclusion

This study documented the prevalence of colibacillosis in layer and broiler birds in the study areas. *E. coli* isolated from colibacillosis affected birds were found to be sensitive to ciprofloxacin, gentamicin, and chloramphenicol which could be used to treat the clinical cases of colibacillosis in poultry. Regular vaccination program along with strict biosecurity measures need to be undertaken to prevent this economically important disease in poultry.

## Authors’ Contributions

MMK and MAI designed the work plan. MAM collected and processed the samples for isolation and identification of bacteria. MAI and MAM carried out PCR and electrophoresis. MAM and MMK interpreted the results and analyzed the data. MAM, MAI and MMK prepared the manuscript. All authors read and approved the final manuscript.
